# Recent advance in the development of tuberculosis vaccines in clinical trials and virus-like particle-based vaccine candidates

**DOI:** 10.3389/fimmu.2023.1238649

**Published:** 2023-11-02

**Authors:** Fangbin Zhou, Dongmei Zhang

**Affiliations:** Department of Tropical Diseases, Naval Medical University, Shanghai, China

**Keywords:** tuberculosis, *Mycobacterium tuberculosis*, vaccine, clinical trials, virus-like particle

## Abstract

Tuberculosis (TB) remains a serious public health threat around the world. An effective vaccine is urgently required for cost-effective, long-term control of TB. However, the only licensed vaccine Bacillus Calmette-Guerin (BCG) is limited to prevent TB for its highly variable efficacy. Substantial progress has been made in research and development (R&D) of TB vaccines in the past decades, and a dozen vaccine candidates, including live attenuated mycobacterial vaccines, killed mycobacterial vaccines, adjuvanted subunit vaccines, viral vector vaccines, and messenger RNA (mRNA) vaccines were developed in clinical trials to date. Nevertheless, many challenges to the successful authorization for the use and deployment of an effective tuberculosis vaccine remain. Therefore, it is still necessary and urgent to continue exploring new vaccine construction approaches. Virus-like particles (VLPs) present excellent prospects in the field of vaccine development because of their helpful immunological features such as being safe templates without containing viral nucleic acid, repetitive surface geometry, conformational epitopes similar to natural viruses, and enhancing both innate and adaptive immune responses. The marketization process of VLP vaccines has never stopped despite VLP vaccines face several shortcomings such as their complex and slow development process and high production cost, and several VLP-based vaccines, including vaccines against Human papillomavirus (HPV), Hepatitis B Virus (HBV) and malaria, are successfully licensed for use at the market. In this review, we provide an update on the current progress regarding the development of TB vaccines in clinical trials and seek to give an overview of VLP-based TB vaccine candidates.

## 1 Introduction

Tuberculosis (TB) is a huge infectious disease caused by *Mycobacterium tuberculosis* (*M. tb*). Over the last decades, several million people lost their lives to TB-related illnesses, ranking over that of malaria and human immunodeficiency virus (HIV) combined (1). While considerable research has been conducted on its interventions and some success has been achieved in some regions around the world, in general, the targets of the UN’s Sustainable Development Goals (SDGs) and the END TB STRATEGY still face huge challenges. Apart from the negative effect of the coronavirus (COVID-19) pandemic, which makes the efforts to end TB more pressing, it is critical to gradually remove the potential threat from people with latent tuberculosis infection (LTBI), which accounts for about one-quarter of the global population. As the World Health Organization (WHO) estimates, about 10.6 million cases acquire TB, and 1.6 million die from this disease in 2021, including 0.19 million concurrent infections with HIV (2). Furthermore, the spread of multidrug-resistant tuberculosis (MDR-TB) and extensively drug-resistant tuberculosis (XDR-TB) makes it more difficult and intractable to control TB effectively.

An effective vaccine is urgently required to achieve the goal of ending TB. Even though most widely used and time-tested, Bacillus Calmette-Guerin (BCG), the only licensed vaccine, is limited to prevent TB. Its efficacy against several aggressive childhood forms of TB such as meningeal and disseminated TB is well recognized, but highly variable at all ages against pulmonary TB (PTB) remains a major concern. Currently, 17 TB vaccines are under active evaluation in clinical trials worldwide, which can be divided according to their design routes into live attenuated mycobacterial vaccines, killed mycobacterial vaccines, adjuvanted subunit vaccines, viral vector vaccines, and messenger RNA (mRNA) vaccines (3, 4). The above types of vaccines have their advantages and disadvantages. Live attenuated vaccines generally produce long-lasting immunity and do not require an adjuvant. Nonetheless, they exhibit several drawbacks, such as high feasibility costs, manufacturing difficulties, and the risk of causing autoimmune, allergic reactions, or disease in individuals with HIV infection or other immune compromises. The production process of inactivated mycobacterial vaccines is mature and easy, however, they might not be perfect, leading to the reversion of attenuated forms to a pathogenic form, as happened in the “Lübeck disaster” where 72 BCG-vaccinated infants out of 252 died after developing clinical or radiological signs of TB (5). Recombinant subunit vaccines can face limitations concerning immunogenicity since they usually consist of several *M.tb* immunogens. However, they suffer the shortcoming of being focused on a narrow set of antigens characterized by suboptimal activity and require suitable immunostimulatory adjuvant or delivery systems to enhance their immunological response. Viral vector vaccines have several advantages as follows: (1) they can carry genes encoding large antigenic fragments; (2) they can induce high levels of both humoral and cellular immune responses; (3) they do not require adjuvants to enhance their immunological response. The major disadvantages of viral vector vaccines include: (1) the pre-existing neutralizing antibodies against the viral vector might limit the booster vaccination strategies; (2) some viral vectors may not be suitable for use in immunocompromised individuals. Since the overwhelming success of mRNA vaccines in defeating COVID-19, mRNA vaccines with lipid nanoparticle delivery systems have raised great interest (6–8). The benefits of mRNA involve it can be extensively engineered for enhanced *in vivo* stability and antigen production no risk of host genomic integration and can stimulate better immune response compared with live virus and DNA vaccines. However, currently, mRNA vaccines require a cold chain for delivery and are expensive to make although manufacturing costs will surely fall in response to demand (9). Alternatively, virus-like particles (VLPs) present excellent prospects in the field of vaccine development as a result of their beneficial immunological features, including good safety without containing viral nucleic acid, repetitive surface geometry, mimicking the size and structure of original viruses and enhancing both innate and adaptive immune responses (10, 11). Since the discovery in the 1980s that the capsule proteins of polyomaviruses can self-assemble into VLP, the research on VLP vaccines has been rapidly developed. Nowadays several VLP-based vaccines, including vaccines against Human Papilloma Virus (HPV), Hepatitis B Virus (HBV), and malaria, are successively, successfully licensed in use (12). Additionally, Human VLP vaccines for influenza virus (IV), HIV, and Ebola virus (EBoV) are also under development (13). However, it is noted that in the field of vaccine development, the main shortcomings of VLP vaccines are their complex and slow development process and high production cost, which are manifested in the following aspects: (1) construction and cloning of viral structural genes. The construction of VLP requires not only the gene sequence of the corresponding virus but also the gene of the target antigen that can be inserted into the gene of the vector capsid protein without affecting the self-assembly of the VLP. (2) screen the appropriate expression system. Common VLP expression systems include Escherichia coli (*E. coli*), yeast, insect cells, etc. Depending on the expression system, there may be different risks, such as low expression, expensive production, etc., so the appropriate expression system needs to be selected carefully. (3) the purification process of VLP involves the steps of cell fragmentation, isolation and purification, sterilization, and filtration, which will face some problems such as VLP degradation, VLP structure destruction, and difficult removal of host nucleic acid. (4) assembly efficiency: Some VLP may need to be depolymerized and reassembled *in vitro* to improve the stability, homogeneity, and immunogenicity of the particles, which has strict requirements for the process. (5) after the structural protein is assembled, whether the ideal structure of the VLP is formed needs to be identified by identification. In this review article, we discuss recent advances in the development of TB vaccines in clinical trials and VLP-based TB vaccine candidates.

## 2 Putative mechanisms of vaccine-induced protective immunity against TB

**Figure 1:**
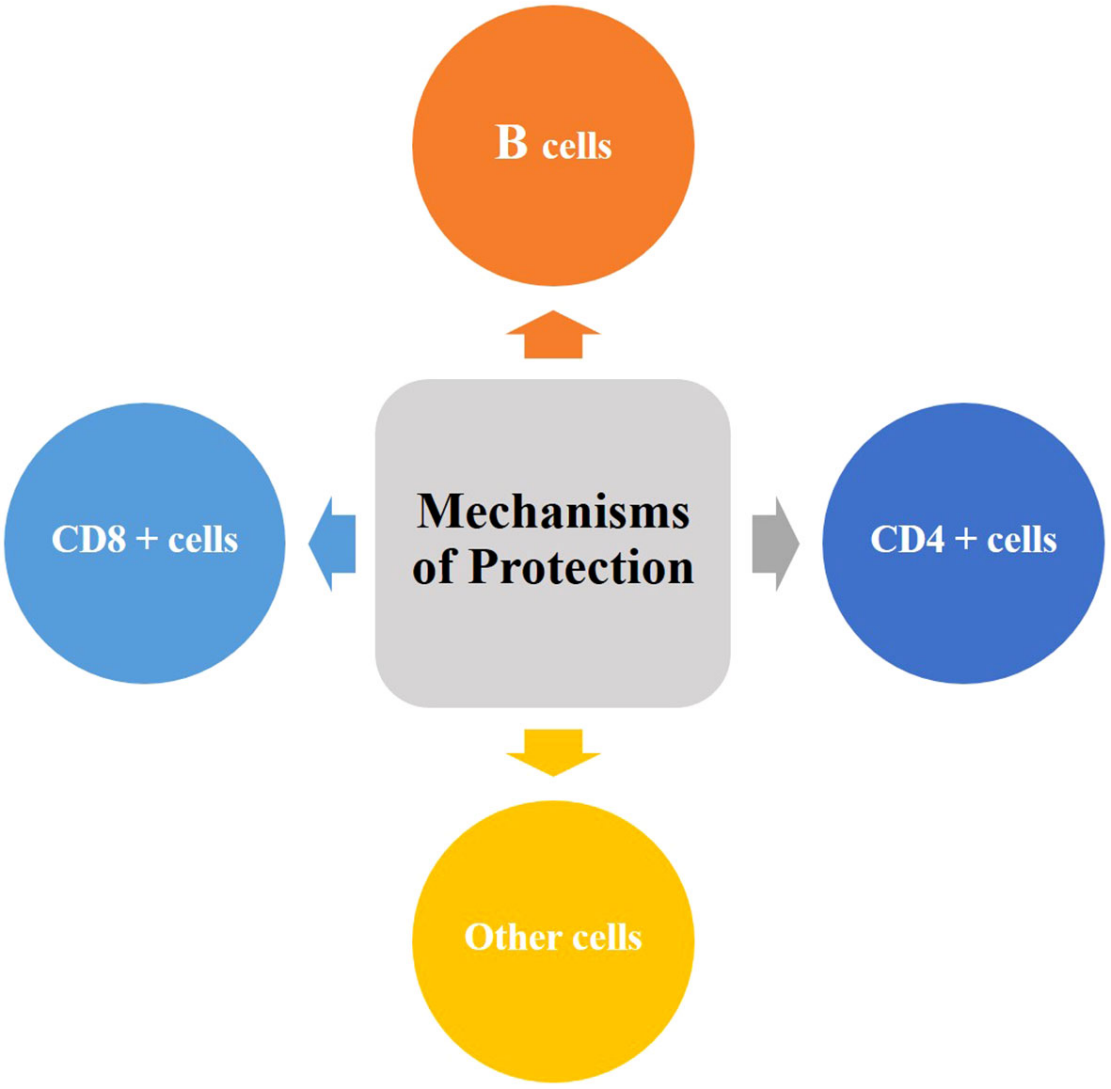
Putative mechanisms of vaccine-induced protective immunity against TB. An ideal TB vaccine will possibly require to engage multiple mechanisms and should aim to elicit a balanced immune response involving CD4 + T cells, CD8 + T cells, B cells and other immune cells such as NK cells and unconventional T cells.

Vaccination mainly aims to establish long-lived and efficient immune memory, such that *M. tb* infection can be controlled rapidly (14). Multiple immune mechanisms engaged by TB vaccines elicit immune responses involving CD4 + T cells, CD8 + T cells, B cells and other immune cells such as NK cells and unconventional T cells (as shown in **Figure 1**). Of these, CD4+ T cells are generally thought to be essential to control TB. For protection against TB, CD4+ T cell differentiate into four main characteristics (15): (1) T central memory (T_CM_) cells that are predominantly found in the lymphoid organs and maintain a high proliferative capacity; (2) T effector memory (T_EM_) cells that are differentiated from T_CM_ cells upon antigen re-exposure and mainly present in circulation and peripheral sites; (3) T tissue-resident memory (T_RM_) cells that a proportion of T_EM_ cells subsequently remains in the lung; (4) T effector (T_EFF_) cells that are newly recruited to arrive after infection. T stem cell memory (T_SCM_) cells (16) and T_CM_ cells, rather T_EM_ cells and T_EFF_ cells, play a central role of the longevity of the immune response for a chronic *M. tb* infection because their proliferative potential can maintain the supply of tissue-homing T cells. It is suggested that *M. tb* infection appears to preferentially drive T cell differentiation toward late-stage T_EM_ cell and T_EFF_ cell responses while the resulting T cell responses seem to be dominated by less differentiated T_CM_ cell responses after TB vaccine administration (14). Achieving long-lived protective immunity by vaccination may require the establishment of a careful balance between *M. tb*-specific T cell differentiation into either T_CM_ or T_EM_ cells.

The role of vaccine-induced CD8+ T cells remains unclear. In a non-human primate (NHP) study, depletion of CD8+ cells led to a significant decrease in compromised BCG vaccine-induced immune control of *M. tb (* 17). However, in mice, vaccine-induced CD8+ T cells failed to recognize *M. tb*-infected macrophages or affect *M. tb* proliferation in animal infection studies even there were very high numbers of CD8+ T cells that were specific for antigens involved in protective immunity (18). Furthermore, several studies have indicated that TB vaccine-induced CD8+ T cells responses had no impact on protection against PTB (18, 19). Taken together, whether TB vaccine-induced CD8+ T cells has a role of protective immunity against TB still remains to be investigated.

It is speculated that the conventional T cell-mediated measurements may be not sufficient to provide enough protective immunity against TB, and previously unrecognized immune mechanisms may contribute to this (20). Substantial experimental evidence that long-term immunity against *M. tb* is cell- mediated led to the notion that B cells and antibodies play little role in protection against TB (21). Nevertheless, there is mounting evidence against this viewpoint (22, 23). two independent reports indicated that antibodies may contribute to protection in some individuals who are still healthy despite long-term, heavy *M. tb* exposures (24, 25). Further, high levels of antigen-specific IgA in bronchoalveolar lavage fluid were found to be associated with protection against *M. tb* infection and disease in a rhesus macaque model of pulmonary BCG vaccination (26). Other immune cells such as vaccine-elicited or trained innate lymphoid cells, unconventional T cells, NK cells, and TRM cells at submucosa may act as sensory cells, recruit memory T cells and early effectors to contribute to abortion of *M. tb* infection (27). To achieve control of *M. tb*, an ideal vaccine should aim to elicit a comprehensive immune response involving that encompasses humoral and cell-mediated immunity as well as multiple-functional immune cells.

## 3 BCG

The BCG vaccine, prepared from live attenuated *Mycobacterium bovis*, is currently the only TB vaccine licensed in clinical (28). BCG is the most widely used vaccine in history and more than 4 billion doses have been administered since the first vaccination in 1921 (29). Multiple studies have exhibited that vaccination with BCG is effective against severe and extrapulmonary forms of pediatric TB (30). Infants and young children can be protected from developing pulmonary and extrapulmonary up to approximately 10 years of age, and even as long as 50–60 years following infant vaccination (31, 32). However, BCG fails to protect adolescents and adults against PTB with its vaccine efficacy ranging from 0% in South India to 87% in the UK (33). Two large, cluster-randomized clinical trials of BCG revaccination in Malawi and Brazil showed no efficacy against TB (34, 35). Nevertheless, previously unrecognized potential of BCG for protective immunity against PTB has been investigated (36, 37). A phase 2b clinical trial showed that BCG revaccination had acceptable safety and induced robust, multifunctional BCG-specific CD4+ T cells (38). Another clinical trial also suggested that BCG revaccination was immunogenic and reduced the rate of sustained QFT conversion, with an efficacy of 45.4% (P=0.03) (39). Furthermore, it was evident that T helper Th 1 and Th17 cells were essential for host protection against TB and BCG revaccination significantly boosted antimycobacterial Th1/Th17 responses in IGRA+ and IGRA– subjects (40). Despite the widespread use of BCG, there are still nearly 25% of the population with LTBI worldwide (41), and 3‰ people are multidrug-resistant latent infection (42). 5-10% of LTBI will develop into TB at some point in the future, thus becoming a potential huge source of recurrent TB infection and an important obstacle to eliminating TB. BCG cannot prevent the progression of LTBI to active TB (43), although several studies have reported that BCG vaccination could provide 20–75% reduction of LTBI risk in children and young adults (44, 45), and BCG vaccination associated with high Neutrophil-to Lymphocyte Ratio (NLR) might have protective effects against LTBI in patients with renal failure or transplant (46). Studies of BCG revaccination in adolescents have not consistently displayed a protective effect against TB and BCG seems to be more efficacious in low-TB-incidence areas farther from the equator (47). One potential explanation for this effect may be that BCG preferentially induces a T effect memory (T_EM_) response, which does not last long enough or is easily consumed following chronic infection or repeated exposure to mycobacterium in these areas (48, 49). Another underlying limitation of BCG is the heterogeneity of the strains of BCG used, each of which has evolved in different regions of the world due to diverse mycobacterial culture conditions. It is not yet clear whether the diversity within and between strains leads to different efficacies of BCG in clinical trials, but these strain differences produce different immune responses in humans and inconsistent protective efficacies in animal models (50, 51). Therefore, the heterogeneity of BCG strains may have an impact on new BCG boosting or supplementation strategies. These limitations associated with the BCG vaccine call for optimization of BCG as well as urgent development of novel and improved vaccine candidates.

## 4 TB vaccine candidates in clinical evaluation

**Figure 2:**
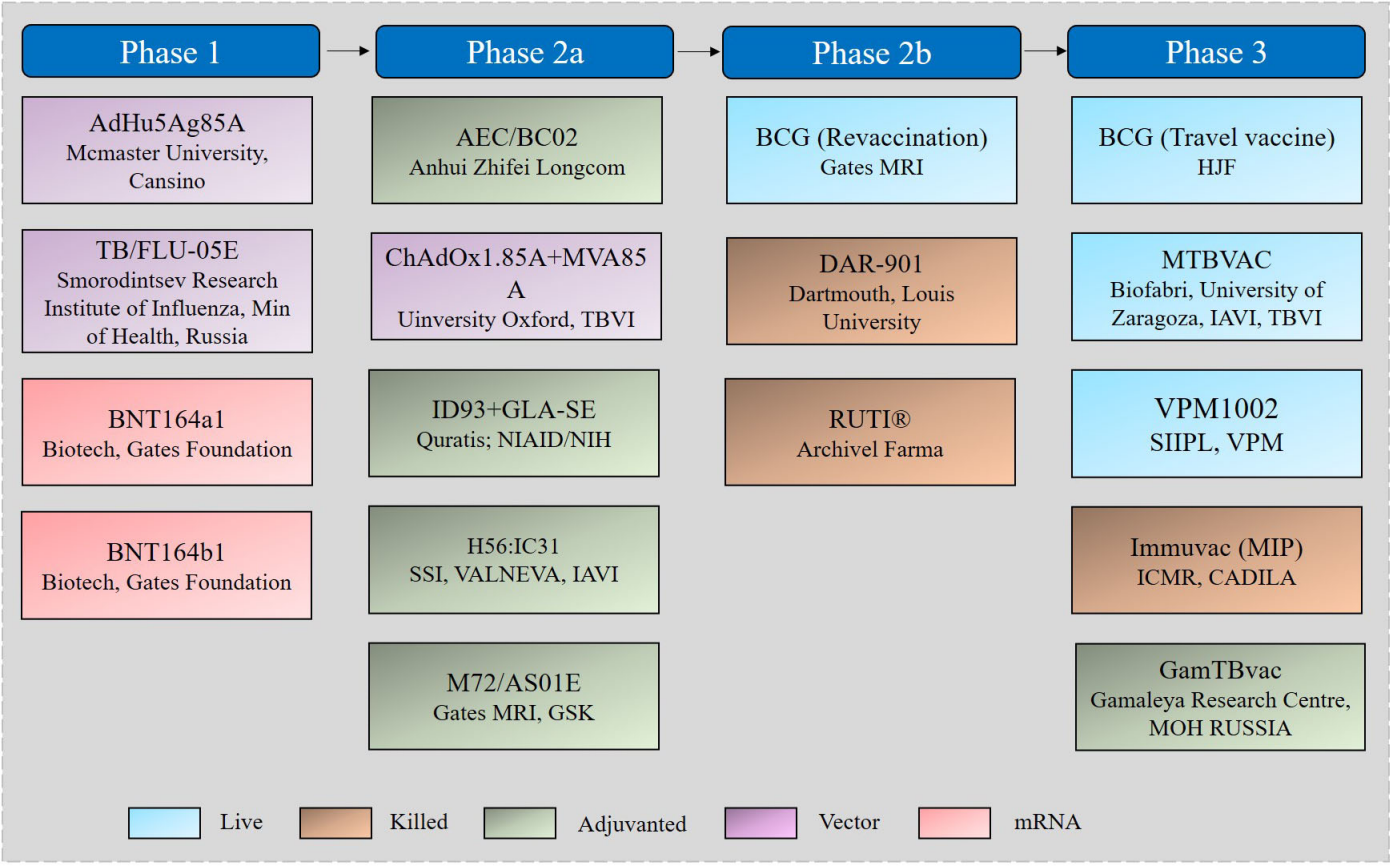
TB vaccine candidates in the clinical pipeline.

Approaches to improve TB vaccination mainly follow one of two strategies: optimization of the current BCG vaccine or development of novel vaccines such as live attenuated or inactivated, subunit, vectored, and mRNA vaccines (52). Nearly 20 TB vaccine candidates have entered the stage of clinical evaluation, 17 of which are currently in clinical trials (**Figure 2**) (4).

### 4.1 Live attenuated mycobacterial vaccines

Live attenuated mycobacterial vaccines were originally designed as priming vaccines to replace or optimize BCG to protect infants and young children against TB but now are being developed to provide post-exposure protection against PTB in adults. A phase 2b clinical trial indicated that BCG revaccination was associated with a higher rate of IGRA reversion, possibly indicative of a protective immune response, and potentially leading to *M. tb* clearance (39). To generate data that can potentially support policy change for BCG (revaccination), the Gates MRI BCG ReVax trial was conducted and AJVaccines’ BCG (*Danish 1331*) was compared with saline placebo for the prevention of sustained IGRA conversion (initial conversion and IGRA-positive 3 and 6 months post initial conversion), as a proxy for sustained *M.tb* infection. BCG (Travel vaccine) is prepared from a live attenuated BCG Tokyo 172 strain supplied by Japan BCG Lab and its phase 3 trial aims to evaluate if a single dose of pre-travel vaccination with BCG can reduce TB infection when given to adults traveling to countries with a high burden of TB. At present, much attention has been paid to developing recombinant BCG (rBCG) vaccine candidates as a potential replacement for BCG since live vaccines elicit a more diverse immune response (4) and the strategies to improve the efficacy of rBCG include deleting genes from BCG and inserting mycobacterial encoding genes into the BCG genome. VPM1002 is currently the only rBCG vaccine in clinical trials and was designed via deleting a urease subunit coding gene in BCG DNA and simultaneously adding of a listeriolysin gene. Phase 1 and 2a clinical trials in healthy infants and adults showed that VPM1002 had good safety and high immunogenicity (**Table 1**) (53, 54). A phase 2b trial in HIV-exposed or -unexposed infants also demonstrated its better safety compared with BCG (55, 56). A series of phase 3 trials were currently undergoing to evaluate VPM1002 to prevent TB disease, TB recurrence, or *M. tb* infection or sustained infection. MTBVAC, developed from the MT103 clinical isolate, is currently the only live attenuated mycobacterial vaccine candidate, with the deletions of two virulence genes *phoP* and *fadD26*. In a preclinical study, it was noted that there was no difference in disease progression between rhesus macaques challenged with an ultra-low dose of *M. tb* Erdman after being vaccinated with BCG or MTBVAC. However, animals vaccinated with MTBVAC outperformed those vaccinated with BCG in reducing lung pathology and extrapulmonary bacterial loads at 16 weeks after infection (57). The first human clinical trial was thus conducted and has proven its favorable safety, and a phase 2 trial conducted in South Africa also showed its acceptable reactogenicity in adults and neonates (55). A phase 3 trial to demonstrate the safety, immunogenicity, and improved efficacy of MTBVAC is undergoing in HIV-uninfected infants born to HIV-infected and HIV-uninfected mothers. For live attenuated strains, safety is one of the most important assessment indicators. The development of the rBCG vaccine AERAS-422, which highly expresses the *M. tb* antigens Ag85A, Ag85B, and Rv3407, was terminated just because two of eight healthy participants had an adverse reaction to shingles (58). Similarly, further clinical evaluation of rBCG30 has been discontinued (59).

**Table 1:** Characteristics of live attenuated and killed TB vaccines in clinical trials.

Vaccine type	Vaccine Candidates	Mycobacterial strains	Modification	Clinical stages
Live	VPM1002	*Mycobacterium bovis BCG-Prague*	addition of a listeriolysin gene, deletion of a urease gene	Phase 3
	MTBVAC	*M. tuberculosis*	Deletions of virulence genes phoP and fadD26	Phase 3
	BCG (Travel vaccine)	*BCG (Tokyo 172)*	–	Phase 3
	BCG (Revaccination)	*AJVaccines’ BCG (Danish 1331)*	–	Phase 2b
Inactivated	M.*vaccae*	*Mycobacterium vaccae*	Heat-killed	Phase 3 (completed, data not published)
	RUTI	*Mycobacterium indicus pranii*	Heat-killed	Phase 2b
	MIP	*Mycobacterium obuense*	Heat-killed	Phase 3
	DAR-901	*Mycobacterium kyogaense* sp. *nov.*	Heat-killed	Phase 2b

### 4.2 Inactivated mycobacterial vaccines

At present, RUTI MIP (*Mycobacterium indicus pranii*), and DAR-901 are the only three therapeutic TB vaccines in clinical trials. Among them, RUTI is an *M. tb* standard strain H37Rv grown under low oxygen partial pressure, low pH, and low nutrition. It is crushed and detoxicated by Triton X-114 and embedded in liposomes. It contains semi-purified and detoxicated fragments of *M. tb* and can express many latent antigens (60). In 2008, a phase 1 clinical trial was conducted to evaluate the safety and immunogenicity of RUTI as a potential therapeutic vaccine in IGRA-negative subjects and found that adverse reactions were positively correlated with dose, which aroused researchers’ concern about the safety of high-dose vaccine injection. At present, a phase 2 clinical trial to assess RUTI as an adjunctive therapy for MDR-TB is underway (60, 61). MIP is a heat-killed vaccine derived from a non-pathogenic bacterium *Mycobacterium indicus pranii*, which was developed by All India University of Medical Science. MIP was initially licensed as an adjunct to chemotherapy for leprosy patients in India, and subsequently, MIP has successively completed phase 1-3 clinical trials of immunotherapy in patients with retreated TB. It was suggested that MIP had a clear effect on *M. tb*, but another phase 3 clinical trial found that MIP was not effective as an adjunct to antituberculosis therapy in patients with TB pericarditis. DAR-901, prepared by *Mycobacterium obuense*, can be used to enhance the immunization of the BCG inoculant population after heat inactivation (62). A phase 1 clinical trial showed that DAR-901 has good tolerance and immune response (63). A phase 2b trial conducted in Tanzania indicated that DAR-901 had good safety and tolerance among BCG-immunized adolescents but was unable to prevent IGRA conversion (64). It is also under evaluation in HIV-infected TB patients as a therapeutic vaccine.

### 4.3 Adjuvanted subunit vaccines

Compared with the above mycobacterial whole cell-derived vaccines, the development of adjuvanted subunit TB vaccines relies heavily on the identification of novel TB antigens involved in protective immunity and the selection of appropriate adjuvant systems (65). Five recombinant subunit vaccines, including M72/AS01E, H56:IC31, ID93+GLA-SE, GamTBvac, and AEC/BC02, are currently in clinical trials, and 11 mycobacterial antigens were used by these vaccines in different combinations and formulations (**Table 2**). M72/AS01E, one of the most promising vaccine candidates under development, is constructed through the fusion of the highly immunogenic *M. tb* proteins Rv1196 and Rv0125, combined with the adjuvant AS01. A series of phase 2 clinical trials completed in India, South Africa, and other regions showed that M72/AS01E was safe and had good immunogenicity in different populations (66). In particular, an earlier phase 2 clinical trial found that the protective efficacy of M72/AS01E in patients with myco-positive TB was up to 54.0%, which meets the WHO’s requirement that the protection rate of adult TB vaccines is not less than 50%. However, the protective effect was reduced to 27.7% in patients with myco-negative TB. One year later, after three years of follow-up, the final results of a phase 2b clinical trial indicated that the vaccine efficacy at month 36 was 49.7% (67). However, it is noted that even if M72/AS01E is proven to be reliable in larger populations, TB control cannot be based on M72/AS01E alone. Compared with the earlier H1:IC31 vaccine (Ag85B-ESAT6 fusion protein), the latency antigen Rv2660c was added to H56:IC31, and its immune effect was significantly improved (68). In 2019, a completed phase 2 clinical trial in South Africa showed that H56:IC31 could stimulate the body to produce a sustained immune response and exhibited good safety and tolerance in adults with or without *M. tb* infection (69). ID93:GLA-SE is prepared with four *M. tb* antigens, namely Rv1813, Rv2608, Rv3619, and Rv3620, combined with the GLA-SE adjuvant (70). A phase 2a clinical trial completed in 2021 showed that ID93:GLA-SE induced a specific pluripotent CD4+ T-cell response to the antigens in adults with completed treatment for TB (71). Additionally, a durable antibody response, producing IgG1 and IgG3 subclasses, was observed, with only routine side effects such as mild induration and erythema. GamTBVac uses a novel vaccine formulation, in which three *M. tb* antigens, namely Ag85A, ESAT6, and CFP-10, are fused with a dextran-binding domain and formulated with an adjuvant (TLR9 agonist) consisting of a Dextran 500 kDa and DEAE-Dextran 500 kDa core covered with CpG oligonucleotides (72). Phase 1 and 2 clinical trials in Russia showed that GamTBvac had favorable safety and immunogenicity (73, 74). Such a vaccine formulation with excellent safety, well-defined molecular composition, directed immunity without vector mediation, and a slow antigen release effect that induces a durable response has attracted much attention. However, this construction method also has some limitations, such as the use of multiple antigens and the complexity of configuration mode will bring great challenges to good manufacturing practice (GMP) and evaluation. AEC/BC02 is made of *M. tb* antigens Ag85B and ESAT6-CFP10, combined with an adjuvant BC02 (75, 76). Two phase 1 clinical trials to assess its safety in adults have been completed, but the results have not been published yet. A phase 2a clinical trial to evaluate the safety, tolerability, and immunogenicity of AEC/BC02 in patients 18 years and older with LTBI is underway.

**Table 2:** Characteristics of subunit and viral vector TB vaccines in clinical trials.

Vaccine type	Vaccine name	Antigens	Formulation or vector	Clinical stages
Subunit	M72/AS01E	Rv1196和Rv0125	AS01	Phase 2b
	H56:IC31	Ag85B、ESAT6 and Rv2660c	IC31	Phase 2b
	ID93+GLA-SE	Rv1813、Rv2608、Rv3619 and Rv3620	GLA-SE	Phase 2a
	GamTBvac	Ag85A,ESAT6 and CFP-10	CpG ODN	Phase 3
	AEC/BC02	Ag85B, ESAT6 and CFP10	BC02	Phase 2a
Viral vector	AdHu5Ag85A	Ag85A	Ad5	Phase 1
	TB/Flu-05E	NS1, TB10.4 and HspX	recombinant attenuated influenza vector (Flu/THSP)	Phase 1
	ChAdOx185A+MVA85A	Ag85A	ChAdOx1, MVA	Phase 2a

### 4.4 Viral vector vaccines

Viral vector vaccines are one of the most useful methods to induce humoral and cellular immunity against TB.ChAdOx185A+MVA85A, AdHu5Ag85A, and TB/Flu-05E are currently three viral vector vaccine candidates in clinical trials. MVA85A was the first viral vector vaccine licensed in clinical evaluation (77, 78). Despite being good safety and immunogenicity in different populations in early trials, MVA85A was proven not to be effective in phase 2b clinical trials (58, 79). The first clinical trial conducted in South Africa indicated that boosting with MVA85A could not significantly improve the ability to prevent TB infection or the onset of TB compared to BCG in BCG-vaccinated infants (77). In a second efficacy trial conducted in HIV-infected people, MVA85A also showed no protective effect, although it enhanced Ag85A-specific Th1 response (79). There are multiple reasons for MVA85A setback, including the use of a single antigen, immunosuppression in people with HIV infection, and lower Ag85A expression after TB infection. However, the researchers still haven’t given up. They combined MVA85A with a monkey adenovirus vaccine, ChAdOx185A, both of which express Ag85A. In a phase 1 trial carried out in adults vaccinated with BCG, ChAdOx185A was used alone or as part of the MVA85A booster immunization strategy (80). Additionally, a phase 2a clinical trial in Uganda was underway in adults and adolescents in 2019 (81). AdHu5Ag85A, formerly Ad5Ag85A, is a recombinant adenovirus type 5 vector that has been engineered to express Ag85A and is employed to enhance protection against TB by boosting BCG (82). In a phase 1 clinical trial, the tolerance and immunogenicity of AdHu5Ag85A were well tested and a more significant immunogenic response was observed in previously BCG-vaccinated volunteers compared with subjects unvaccinated with BCG (82). In a phase 2a clinical trial, it was proven that aerosol delivery, but not an intramuscular injection, of AdHu5Ag85A, induces respiratory-mucosal immunity in humans (83). TB/Flu-01L is a replication-deficient influenza virus A expressing ESAT-6 antigen. The assessment of the safety of TB/Flu-01L has been well completed in a phase 1 clinical trial in 2023 and a phase 2 clinical trial has not yet been planned. TB/Flu-04L is another viral vector vaccine that is based on the attenuated influenza strain Flu NS106 encoding *M. tb* antigens EAST-6 and Ag85A (84). In a phase 1 trial in healthy BCG-vaccinated, QFT-negative adults in Kazakhstan, the safety and immunogenicity of TB/Flu-04L have been verified. phase 2a trials of TB/FLU-04L are postponed until additional preclinical reproductive toxicology studies are done. Compared with TB/Flu-01L and TB/Flu-04L, which were both removed from the TB vaccine pipeline, TB/FLU-05E was a mucosal TB vaccine candidate based on recombinant attenuated influenza vector (Flu/THSP) co-expressing truncated NS1 protein NS1(1–124) and a full-length TB10.4 and HspX proteins of *M.tb* within an NS1 protein open reading frame. Preclinical trials indicated that TB/FLU-05E was safe and stimulated a systemic TB-specific CD4+ and CD8+ T-cell immune response to provide protection against TB in mice and guinea pigs (85, 86).

### 4.5 mRNA vaccines

During the COVID-19 pandemic, mRNA vaccines, developed by Moderna and Pfizer/BioNTech, were authorized and licensed for use in humans for the first time (87, 88). Inspired by the tremendous success of COVID-19 mRNA vaccines, the development of mRNA vaccines against other infectious diseases such as monkeypox (mpox) and TB has received unprecedented attention (9, 89). In 2004, a protective effect of RNA vaccination against TB was demonstrated while its protection was less than that obtained with BCG (90). In 2023, BioNTech initiated a randomized, controlled, dose-finding phase 1 clinical trial of BNT164, the first mRNA vaccine candidates targeting TB, in partnership with the Bill and Melinda Gates Foundation. The clinical trial will evaluate the safety, reactogenicity, and immunogenicity of BNT164a1 and BNT164b1 against TB.

## 5 Recent advances in the development of VLP-based TB vaccines

### 5.1 The concepts and characteristics of VLPs

VLPs are hollow particles that contain one or more self-assembled, structural proteins of a virus but do not contain viral nucleic acids, commonly known as pseudoviral particles (11). With the development of molecular biology technologies, the chimeric VLPs obtained by covalent actions or covalent modifications also belong to the category of VLPs. As a new type of subunit vaccines, VLPs have several advantages as follows, firstly, compared with a single protein or peptide, the conformational epitopes of VLPs are more similar to that of original viruses, thus significantly enhancing the level of immune response. Secondly, without affecting the structure of VLPs, some targeted amino acid sequences can be inserted or deleted in accordance with requirements, and artificial modifications can be made to construct chimeric VLPs. In addition, VLPs can also be developed as carriers to deliver some small molecules or drugs, or as carriers for the delivery of DNA or RNA vaccines to improve their immune efficacy or for gene therapy (10). Nevertheless, the insertion of the sequence of target antigens should not significantly influence the formation of VLPs, and the appropriate expression system should be selected carefully. Moreover, a proper purification method should be explored to avoid common problems such as VLP degradation, VLP structure destruction, and difficult removal of host nucleic acid. whether the ideal structure of the VLP is formed also needs to be identified after assembly efficiency is assessed.

Based on the structural characteristics of native viruses, VLPs can be generally divided into two main categories, non-capsulated and capsulated VLPs. Non-capsular VLPs are usually made up of one or more self-assembled capsid proteins and don’t contain any host cell components. The major capsid protein L1 of HPV and VP2 protein of porcine parvovirus (PPV) are two typical examples. The structures of capsulated VLPs are more complex, which contain cell membrane components derived from host cells. The capsular components cover the surface of the VLPs and exhibit similar structures and functions to native viruses, such as HBV, HIV, and hepatitis C virus (HCV), etc.

Various expression platforms, including eukaryotic, prokaryotic, and cell-free systems derived from eukaryotic or prokaryotic expression systems, can be employed for producing VLPs. Eukaryotic expression systems include yeast systems, mammalian cell systems, baculovirus/insect cell (B/IC) systems, and plant systems. Prokaryotic systems mainly include *E. coli* systems, while cell-free systems include wheat germ cells and rabbit reticulocyte systems according to the source of raw materials (91). About 70% of reported VLPs are prepared by eukaryotic systems while 30% by prokaryotic systems. In general, the viral proteins produced by mammalian cells and plant cells have the most complete structures and functions, which are conducive to the efficient packaging of VLPs and the maintenance of VLPs activity. *Pichia pastoris*, *Saccharomyces cerevisiae*, and *Saccharomyces polymorphus* are the most widely used yeast cells, which can carry out post-translational modification (PTM) of the expressed proteins to make them have the correct conformations and biological activities. However, it has limited function in post-translational modification, and thus not used for the preparation of capsular VLPs.

### 5.2 VLP-based vaccines against human diseases

There are over 110 viral proteins from 35 viral families that have been demonstrated to be able to self-assemble as VLPs (92). Multiple advantages and ascendant characteristics make VLPs have good prospects in the field of vaccine application. In the early 1980s, the HBV vaccine was developed as the first commercial VLP-based vaccine (93). The researchers identified a non-infectious particle that is an irregular lipoprotein structure rather than a highly repetitive, ordered protein complex but has similar immunogenicity and the ability to induce and neutralize antibodies against native viruses. Subsequently, the VLP-based HPV quadrivalent vaccine Gardasil was approved for clinical use by the Food and Drug Administration (FDA). A large number of clinical trials have shown that Gardasil has strong immunogenicity and significant prevention and control effects on female genital tract diseases caused by HPV (94). In 2012, Xia’s team from Xiamen University successfully developed the world’s first commercial VLP-based hepatitis E virus (HEV) vaccine Hecolin, which is a huge breakthrough in the prevention and control of HEV worldwide (95). Several other VLP-based vaccines, including HIV, HCV, Dengue virus (DENV), EBoV, Marburg virus, and Chikungunya virus (CHIKV), are also in preclinical stages (96).

### 5.3 VLP-based TB vaccine candidates

Recently, the idea of constructing a VLP-based vaccine has also been quietly embedded in the development of TB vaccines (**Table 3**). Influenza A VLPs have been successfully used as vaccine expression platforms, including mammalian cells and B/IC. Hemagglutinin (HA) and neuraminidase (NA) are two major glycoproteins on the surface membrane of influenza A virus. The M1 protein is the main protein that forms the viral coat and is critical for the formation of VLPs. ESAT-6 is a famous antigenic target with a large number of T cell epitopes and has been widely used for TB vaccine research. In Florian’s research, 20 amino acid sequence from ESAT-6 was presented on the antigenic region B of the influenza HA, and an influenza A VLP was generated (97). Humoral immunity after immunizing mice suggested that influenza A VLPs could be capable of presenting foreign TB epitopes as a beneficial platform.

**Table 3:** Characteristics of VLP-based TB vaccine candidates.

Candidates	VLP vectors	Antigens	Expression Systems
HA-ESAT-6	Influenza A HA	ESAT-6	Insect cell-baculovirus
HE6	Hepatitis B HBc	ESAT-6	*E. coli*
FVLP	Hepatitis B HBc	CFP-10	*E. coli*
HPV16L1/Ag85B	HPV16 L1	Ag85B	*Pichia pastoris*

HBV core protein (HBc) is a structural nucleocapsid protein of HBV and can self-assemble into VLPs. Ying et al. employed HBc as an immune platform to enhance the immunogenicity of *M. tb* antigen ESAT-6 (98). ESAT-6 was firstly cloned into the major immunodominant region (MIR) of HBc by fusion PCR, which does not influence particle formation. Recombinant HBc-ESAT-6 (HE6) was then produced in *E. coli* and the formation of VLPs was confirmed by electron transmission microscopy (TEM). The immunogenicity of HE6 was well tested and the results indicated that HE6 immunization elicited both humoral and cellular immunity.

*M. tb* antigen culture filtrate protein 10 (CFP-10) is another excellent antigen used for TB vaccine development, which induces strong T-cell immunity. However, the immune response induced by the sole antigen without an adjuvant is much lower. Thus, Dhananjayan et al. used an HBc VLP as a versatile tool to enhance the immunogenicity of CFP-10 (99, 100). CFP-10 presenting on nano-sized HBc-VLPs were produced in *E. coli* and TEM was used to validate the formation of fusion protein VLPs (FVLP). The challenge experiment results indicated that FVLP induced significantly higher levels of IgG and IgG2a antibodies compared with CFP-10 protein alone, cells from FVLP-immunized mice produced higher levels of IL-2, IFN-γ, and TNF, and splenocytes from animals immunized with FVLP contributed to higher proliferative responses when stimulated by CFP-10 *in vitro*. The above excellent humoral and cellular responses implied the huge potential for HBc-VLPs as a helpful vaccine delivery tool for presenting TB epitopes.

The major capsid protein L1 of HPV type 16 (HPV16L1) contains 505 amino acids in its full length and is capable of self-assembly into VLPs; it is also characterized by three virus-facing hinge regions between beta folds: DE, FG, and HI loops (101). The results showed that the insertion of short foreign sequences in the FG loop did not influence the formation of VLPs. In our study, the VLPs of HPV-16 L1 were used as the carrier to insert the TB antigen Ag85B and construct the chimeric molecule of HPV-16 L1/Ag85B. Western blot confirmed the successful expression of HPV16L1 chimeric Ag85B in *Pichia pastoris*. TEM examination results showed that HPV-16 L1/Ag85B could form particles with a diameter of approximately 50 nm and the immunogenicity of HPV-16 L1/Ag85B was also evaluated (relevant data have not yet been published) (102).

It was noted that a potential disadvantage of the VLP vaccine is that substantial levels of anti-vector antibodies tend to be preexisting in humans, although most inventors of the vaccines demonstrated that vector antibodies do not affect the safety and immunogenicity of VLPs. In my opinion, there are the following points that may affect the T cell response toward the inserted protein epitopes of VLPs. Firstly, make sure that the inserted fragments into vectors contain a dozen T-cell epitopes. Secondly, it is important to confirm that sequentially inserted protein epitopes were presented in chimeric VLPs. We also agree that priming with natural HPV infection or VLP vaccination may generate a T cell response that could limit the T cell response toward the targeted protein epitopes although it is noted that chimeric L1:P18I10/L1:T20 VLPs could simultaneously elicit HPV16- and HIV-1-specific T-cell responses in BALB/c mice (103). In addition, a clinical trial also indicated that despite prevalent preexisting anti-AdHu5 humoral immunity in most of the trial volunteers, little evidence that such preexisting anti-AdHu5 immunity significantly dampened the potency of the AdHu5Ag85A vaccine (104).

Protective immunity against ESAT-6 and CFP-10 may be mediated by mechanisms that depend not only on T cells but also on humoral/antibody responses to ESAT-6 and CFP-10 epitopes on VLPs. However, how can VLP-induced humoral/antibody responses to ESAT-6 and CFP-10 epitopes mediate protective immunity against TB? As a review indicated (20), there are several mechanisms by which antibodies might impact on the response to ESAT-6 and CFP-10 epitopes on VLPs, such as blocking the uptake of *M. tb* by non-professional phagocytes that provide a protective niche for the mycobacteria, targeting uptake by professional phagocytes with high antibacterial capacities and activating of antibacterial mechanisms in phagocytes by stimulation through Fc receptors.

## 6 Conclusions

Despite some advances that have been made in the development of novel TB vaccines, with as many as a dozen TB vaccine candidates in clinical stages, there are still many limitations of current TB vaccines. On the one hand, most vaccines employ a limited pool of immunologically dominant target antigens, mainly from the secreting protein Ag85 and ESAT-6 families. In mice, it has been found that the limitation of antigen availability weakens the Ag85 B-induced protective immunity during chronic infection while the immunity of ESAT-6-specific T cells is restricted as a result of functional exhaustion, highlighting potential challenges in employing these antigens for vaccine development (105). As the antigen-specific natural T cell response of *M. tb* is highly heterogeneous, tens of antigens are required to cover 80% of CD4 T cell responses. However, current vaccine approaches utilizing a few antigens may not induce a sufficient immune response. Conversely, antigens that are poorly recognized during natural infection (referred to as unnatural antigens) may not be fully recognized by the immune system at all during infection, and their role in protection is unclear and worthy of further investigation. The subunit vaccine M72: AS01E is composed of the unnatural antigens Rv0125 and Rv1196, combined with the adjuvant AS01. Phase 1 and 2 clinical trials in non-*M. tb* negative populations have indicated that M72:AS01E has a relatively high T-cell immune induction effect (66). On the other hand, the parameters to evaluate an eligible vaccine are too simple, which pays too much attention to the Th1 immune response effect induced by vaccines. A review study showed that six TB vaccine candidates, including MVA85A, AERAS-402, H1:IC31, H56:IC31, M72/AS01E, and ID93:GLA-SE, evoked highly similar functional properties of memory T cell responses, indicating a lack of diversity among the available TB vaccine candidates. Although an effective Th1-type cell-mediated adaptive immune response characterized by the secretion of IFN-γ and TNF by antigen-specific CD4 T cells is known to be required, it is not sufficient to provide protective immunity against TB. In view of the complexity of TB-host interaction, the mechanism related to immune response to TB is still poorly understood. A too-simple evaluation index of a qualified vaccine will greatly increase the risk of vaccine failure, thus new biological markers of vaccine protective immunity are urgently needed. VLP-based vaccines have gradually become a hotspot in the field of vaccine development due to their numerous characteristics, including, but not limited to, their good safety without risk of infection, and their abilities to mimic the size and structure of original viruses and to display foreign antigens on their surface to enhance the immune response. The marketization process of VLP vaccines has never stopped despite the VLP vaccine’s need to overcome several shortcomings, including their complex and slow development process, and high production cost. At present, some VLP-based vaccines including IV, HBV, HEV, HPV, and malaria, have been successfully licensed at the market (11), and VLP-based vaccines designed for HIV, EBoV, and other infectious diseases are in preclinical development or clinical trials (96). Some advances in the development of VLP-based TB vaccines are also been made (97–100), the key of which lies in finding novel antigens with high immunogenicity and improving the form of vaccine construction to stimulate the immune system. It is believed that novel TB antigenic targets and VLP carriers will be continuously identified and exploited with in-depth research on the relationship between TB and host immunity, and more safe and effective vaccines must be developed to prevent TB in humans in the future.

## Author contributions

FZ conceived, designed, and wrote the manuscript. FZ and DZ revised the manuscript. All authors contributed to the article and approved the final manuscript.
